# HTLV-1-infected CD4+ T-cells display alternative exon usages that culminate in adult T-cell leukemia

**DOI:** 10.1186/s12977-014-0119-3

**Published:** 2014-12-18

**Authors:** Morgan Thénoz, Céline Vernin, Hussein Mortada, Maroun Karam, Christiane Pinatel, Antoine Gessain, Thomas R Webb, Didier Auboeuf, Eric Wattel, Franck Mortreux

**Affiliations:** Université de Lyon 1, CNRS UMR5239, Oncovirologie et Biothérapies, Laboratoire de Biologie Moléculaire de la Cellule, Faculté de Médecine Lyon Sud, Pierre Bénite, France; Centre de Recherche sur le Cancer de Lyon, France Epissage alternatif et progression tumorale, Lyon, France; Institut Pasteur, Unité d’Epidémiologie et Physiopathologie des Virus Oncogènes, Paris, France; SRI International, 333 Ravenswood Avenue, Menlo Park, CA 94025-3493 USA; Université Lyon I, Service d’Hématologie, Pavillon Marcel Bérard, Centre Hospitalier Lyon-Sud, Pierre Bénite, France; Oncovirologie et Biotherapies, Laboratoire de Biologie Moléculaire de la Cellule, UMR5239 CNRS/ENS, Lyon/UCBL/HCL; Ecole normale supérieure de Lyon, 46, allée d’Italie; 69364, Lyon cedex 07, France

**Keywords:** HTLV-1, Adult T-cell leukemia, Alternative splicing

## Abstract

**Background:**

Reprogramming cellular gene transcription sustains HTLV-1 viral persistence that ultimately leads to the development of adult T-cell leukemia/lymphoma (ATLL). We hypothesized that besides these quantitative transcriptional effects, HTLV-1 qualitatively modifies the pattern of cellular gene expression.

**Results:**

Exon expression analysis shows that patients’ untransformed and malignant HTLV-1^+^ CD4^+^ T-cells exhibit multiple alternate exon usage (AEU) events. These affect either transcriptionally modified or unmodified genes, culminate in ATLL, and unveil new functional pathways involved in cancer and cell cycle. Unsupervised hierarchical clustering of array data permitted to isolate exon expression patterns of 3977 exons that discriminate uninfected, infected, and transformed CD4^+^ T-cells. Furthermore, untransformed infected CD4+ clones and ATLL samples shared 486 exon modifications distributed in 320 genes, thereby indicating a role of AEUs in HTLV-1 leukemogenesis. Exposing cells to splicing modulators revealed that Sudemycin E reduces cell viability of HTLV-1 transformed cells without affecting primary control CD4+ cells and HTLV-1 negative cell lines, suggesting that the huge excess of AEU might provide news targets for treating ATLL.

**Conclusions:**

Taken together, these data reveal that HTLV-1 significantly modifies the structure of cellular transcripts and unmask new putative leukemogenic pathways and possible therapeutic targets.

**Electronic supplementary material:**

The online version of this article (doi:10.1186/s12977-014-0119-3) contains supplementary material, which is available to authorized users.

## Background

HTLV-1 causes chronic infection that relies on the persistent clonal expansion of infected CD4^+^ and CD8^+^ T-cells. Reprogramming of gene transcription sustains HTLV-1 viral persistence and ultimately leads to the development of adult T-cell leukemia/lymphoma (ATLL) from a CD4^+^-infected clone in a minority of carriers after a prolonged latency [1]. Transcriptome analyses using microarray technology has provided key insights into the biological processes involved in the pre-malignant and malignant expansion of infected cells [2-14]. HTLV-1 has been found to deregulate the expression of numerous genes involved in key cellular pathways such as the cell cycle, apoptosis, telomeres/telomerase, DNA repair, and immune and inflammatory responses through expression of the *Tax* oncoprotein [2-6].

Recent works have highlighted that in addition to their quantitative effects on gene expression, numerous pathogenic processes, such as persistent viral infections [15] or tumor development [16,17], rely on acquired alternate exon usage (AEU) events. As for other retroviruses, alternative splicing plays a pivotal role in HTLV-1 expression. From its 5’LTR, HTLV-1 transcribes a single polycistronic pre-mRNA that codes for structural and enzymatic proteins required for viral particle production. This pre-mRNA also undergoes multiple alternative splicing events that generate mono-spliced transcripts coding for the regulatory proteins p21Rex, p12 and p13, and double-spliced transcripts coding for Tax, p27Rex and p30 [18,19]. Similarly, minus-strand transcription initiated from the 3’LTR generates spliced and unspliced RNA isoforms of HBZ that synthesize HBZ proteins with distinct properties on cell proliferation [20-22]. How HTLV-1 intervenes in alternative splicing processes is still incompletely understood. It has been reported that the RNA-binding protein Rex regulates viral splicing through interacting with host splicing machinery to inhibit viral RNA splicing and export unspliced and single-spliced transcripts [23-25]. Gene-by-gene analyses have further shown that HTLV-1 may affect alternative splicing of cellular genes including CD44 [26] and IL-6- and IL-2-receptors [27,28], proposing that HTLV-1-induced AEUs might contribute to molecular mechanisms that underlie the clonal expansion and the malignant transformation of infected cells. However, no systematic study has been hitherto conducted to ascertain the extent of alternative splicing modifications upon HTLV-1 infection. Here, by using integrative analysis of exon expression profiles and gene ontology of CD4^+^ T-cells derived from infected individuals with and without malignancy, we show that HTLV-1 induces multiple AEU alterations that unmask new putative leukemogenic pathways and possible therapeutic targets.

## Results and discussion

Comparative microarray analysis of exon expression profiles was performed with three ATLL samples and 12 untransformed CD4^+^ T-cell clones (six infected) derived from HTLV-1-infected individuals with no clinical signs of malignancy. ATLL samples were obtained from patients with an acute form of ATLL (>95% circulating malignant cells). CD4^+^ clones were obtained through cloning by limiting dilution of peripheral blood mononuclear cells (PBMCs) derived from three HTLV-1-infected individuals with tropical spastic paraparesis/HTLV-1-associated myelopathy with a disease duration of 6, 11, and >26 years. This study was conducted according to the principles outlined in the Declaration of Helsinki, and approved by the Institutional Review Board of the Hospices Civils de Lyon (France). As previously described [29,30], CD4^+^ clones were submitted to *ex vivo* culture for one month prior to HTLV-1 screening and RNA extraction. At this time, infected and uninfected cells remained non-immortalized and required IL-2 for continued growth. Given that *in vitro* T-cell activation is known to modify AEU [31,32], exon array analysis of untransformed CD4^+^ clones was carried out with RNAs extracted from unstimulated and phytohemagglutinin (PHA)-stimulated CD4^+^ T-cells (Additional file 1: Table S1). By this approach that take into account the in vitro cell culture, we assumed that significant changes in exon expression between infected and uninfected clones mainly resulted from the global impact of HTLV-1 infection, irrespective of activation status. The microarray data have been deposited *in toto* into the Gene Expression Omnibus database and are available under record number GSE52244.

**Figure 1:**
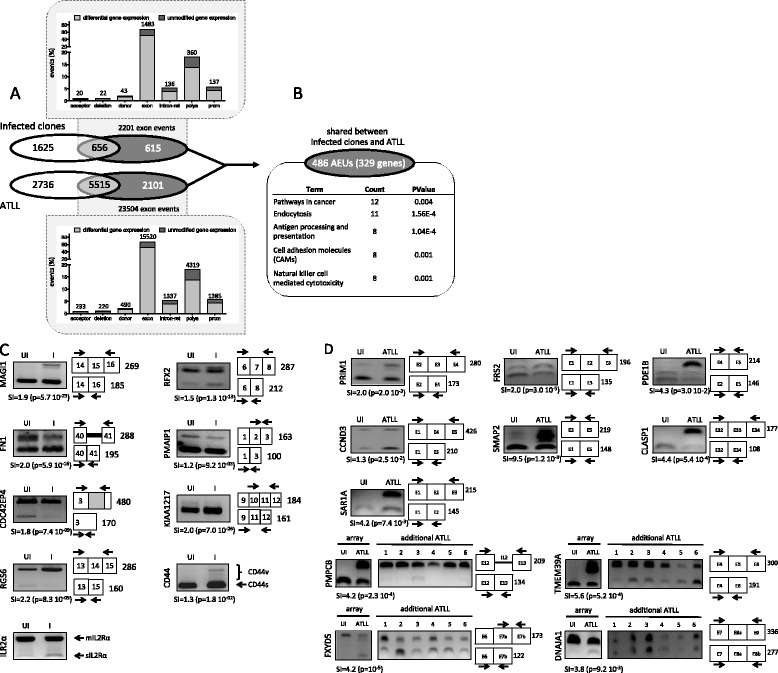
**Distribution of AEU in ATLL cells and cloned CD4** ^**+**^ **T-cells derived from HTLV-1-infected individuals. (A)** *Distribution of quantitative and qualitative HTLV-1 modifications in untransformed HTLV-1-positive CD4* ^*+*^ *clones and ATLL samples.* The Venn-diagrams show the distribution of genes modified at the whole gene expression level (white), AEU (dark grey), or both (light grey) in ATLL samples (bottom) and untransformed infected clones (top; six independent data sets including three PHA-activated and three unactivated clones) versus uninfected clones (six independent data sets including three PHA-activated and three unactivated clones). Graphs present the distribution of each class of AEU annotated according to the FASTERDB database [41]. Acceptor: alternative acceptor site; deletion: exon deletion; donor: alternative donor site; exon: exon skipping; intron-ret: intron retention; polya: alternative polyadenylation site; prom: alternative promoter. The total number of each class of exon event is indicated on the histogram bar. **(B)** *GO analysis of genes (n = 329) presenting common AEUs in untransformed-infected clones and in ATLL samples (compared with uninfected clones).* The complete set of genes featured in microarrays (42304 Ensembl geneIDs) was used as a reference background. Gene annotations are presented in Additional file 5: Table S3. **(C)** *RT-PCR validation of microarray-predicted exon events in CD4* ^*+*^ *T-cells derived from HTLV-1 carriers compared with uninfected T-cell clones.* Exon-specific RT-PCR was performed with pooled RNA samples derived from six infected (I) and six uninfected (UI) clones, thereby reflecting the distribution of splice variants in all clones irrespective of activation status. Numbers indicate the expected band (bp) of PCR products, and the SI and p-value are indicated for each exon event; a SI ≥ 1.2 was considered to be a significant change in exon expression and used for comparisons. **(D)** *RT-PCR validation of microarray-predicted exon events in ATLL cells (array) and exon-specific RT-PCR of six additional ATLL samples compared to uninfected T-cell clones*.

HTLV-1 expression was assessed by quantitative RT-PCR (qRT-PCR) of *Tax* transcripts. In accordance with previous reports, *Tax* transcripts were weakly detected in 2 out of 3 ATLL samples (Additional file 2: Figure S1) [33-35]. In comparison, there was a wide range of *Tax* expression between clones (0.16-2.20; median, 1.2; mean ± standard deviation [SD], 1.172 ± 0.799). From these data, we assumed that HTLV-1 samples exhibited typical features of untransformed- and transformed-infected CD4^+^ T-cells *in vivo*. Computational analyses of 15 exon arrays and annotation of exon events were achieved as detailed in Figure 1. Figure 1A represents the distribution of quantitative and qualitative gene modifications in untransformed HTLV-1-infected CD4^+^ clones and ATLL cells. A total of 18/20 (90%) array-predicted exon usages were validated by exon-specific RT-PCR (Figures 1C and D). Among those, we found alternative splicing changes in *IL2-R* and *CD44* transcripts (Figure 1C). These data confirmed and extended previous observations indicating that IL2-R and CD44 variant isoforms are detected in the PBMC of infected individuals [26,28]. Array-predicted changes in transcript abundance were validated by qRT-PCR for 8/9 (88%) genes (Additional file 3: Figure S2).

To examine the global impact of HTLV-1 on AEU of CD4^+^ T-cells, we investigated AEU in untransformed CD4^+^ T-cells by comparing the pattern of exon expression in infected versus uninfected clones (Figure 1A; Additional file 1: Table S1). Overall, 2201 AEU events were identified in 1271 genes, of which only 656 (51%) were found altered at the level of whole gene expression. This demonstrated that in untransformed cells, HTLV-1 significantly modifies the exon content of multiple transcripts, considering half of these genes were transcriptionally unmodified. Secondly, when ATLL samples were compared with uninfected clones, the overall number of AEU events was found to be 11-fold (23504 versus 2201) higher than that distinguishing HTLV-1-positive from -negative untransformed CD4^+^ T-cells (Figure 1A). In addition to this quantitative difference, the proportion of AEU events found in differentially expressed genes was significantly higher in ATLL samples (72% versus 51% in uninfected clones; p < 0.0001, Pearson’s Chi-squared test; Figure 1A). Together these results indicate that the distribution of AEU alterations was significantly different between ATLL and HTLV-1-positive untransformed cells.

The comparative analysis of AEU between the two categories of infected cells revealed that infected CD4^+^ clones and ATLL samples shared 486 AEUs in 329 genes (p = 1.83 × 10^−21^, Hypergeometric test), suggesting that 22% of AEUs arising at the chronic stage of infection might pertain to ATLL development. The number of AEUs per gene ranged from one to 20 (median, 1; mean ± SD, 1.47 ± 1.42; Additional file 4: Table S2). GO enrichment analysis (DAVID resources, http://david.abcc.ncifcrf.gov/) performed with this set of genes identified several enriched terms that fit well with HTLV-1 infection and related diseases, such as pathways in cancer (fold enrichment [FE]: 2.71), endocytosis (FE: 4.45), antigen processing and presentation (FE: 7.23), cell adhesion molecules (CAMs; FE: 4.56), and natural killer cell-mediated cytotoxicity (FE: 4.46; Figure 1B; Additional file 5: Table S3).

**Figure 2:**
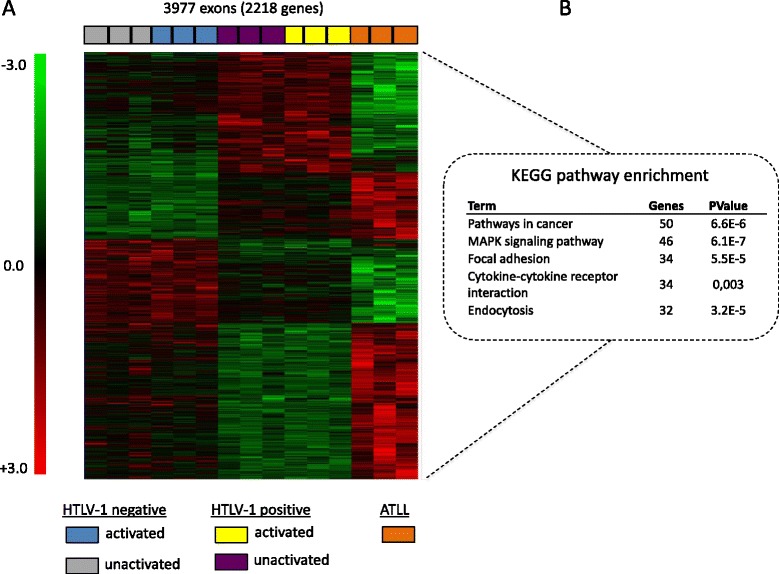
**Exon-based hierarchical clustering.** Hierarchical clustering analysis was performed with Mev4.0 software (http://www.tm4.org/) using the gene-normalized exon intensities. **(A)** The selection of exons useful for differentiating between the 15 cell samples was statistically analyzed using a Kruskal-Wallis ANOVA with p < 0.05 representing statistical significance. **(B)** *GO analysis*. Top pathways are presented. The gene set corresponded to 2000 genes that displayed the highest splicing index (SI) values. The complete set of genes featured in microarrays (42304 Ensembl geneIDs) was used as a reference background. Official gene symbol annotations are presented in Additional file 5: Table S3.

Hierarchical clustering of exon array data showed that alternative expression of 3977 exons (2218 genes) permitted sample gathering in three distinct clusters, including untransformed-uninfected and infected CD4^+^ clones, as well as ATLL samples (p < 0.05, Kruskal-Wallis ANOVA; Figure 2A). For untransformed cells, these exon expression profiles were irrespective of PHA stimulation, implying a direct role of HTLV-1 in these recurrent alternative splicing modifications. ATLL cells exhibited marked changes in AEU compared to untransformed HTLV-1-positive or -negative CD4^+^ clones. Such distribution suggests that some AEU events could represent an ATLL molecular signature. Therefore, we used exon-specific RT-PCR to analyze six additional samples harvested from patients with acute forms of ATLL. Over the 11 events identified in Figure 1D, the expression of 4 AEUs was found to be recurrent in ATLL (Figure 1D). Whether the AEUs shown in Figures 1B, D, and 2A are involved in ATLL development and possess diagnostic and/or prognostic implications deserves further investigation.

A GO analysis was performed to gain insight into the functional significance of the exon expression profiles (3977 exons) that discriminate between the three groups of samples presented in Figure 2A. Significant enrichment was detected for genes involved in important biological processes, such as those related to cancer (50 genes, FE = 1.9; p = 6.6 × 10^−6^), MAPK signaling (46 genes, FE: 2.2; p = 6.1 × 10^−7^), focal adhesion (34 genes, FE: 2.2; p = 5.5 × 10^−5^), cytokine-cytokine receptor interactions (34 genes, FE: 1.7; p = 0.003), and endocytosis (32 genes, FE: 2.2; p = 3.2 × 10^−5^; Figure 2B). Official symbol annotation of genes with AEUs that are enriched in these pathways are presented in (Additional file 6: Table S4). Secondly, GO analysis of AEU events detected in ATLL samples (7616 genes) and untransformed HTLV-1-positive CD4^+^ T-cell clones (1271 genes) showed distinct biological changes (Additional file 7: Figure S3). For example, genes encoding spliceosome components appeared exclusively enriched in ATLL cells, while cell cycle-related genes were found enriched in untransformed and transformed HTLV-1 positive samples. Moreover, AEU ontological enrichments were different from those of differentially expressed genes (Additional file 7: Figure S3). Together, these results suggest that HTLV-1-related transcriptional and post-transcriptional changes might pose distinct functional impacts on untransformed and malignant cells.

**Figure 3:**
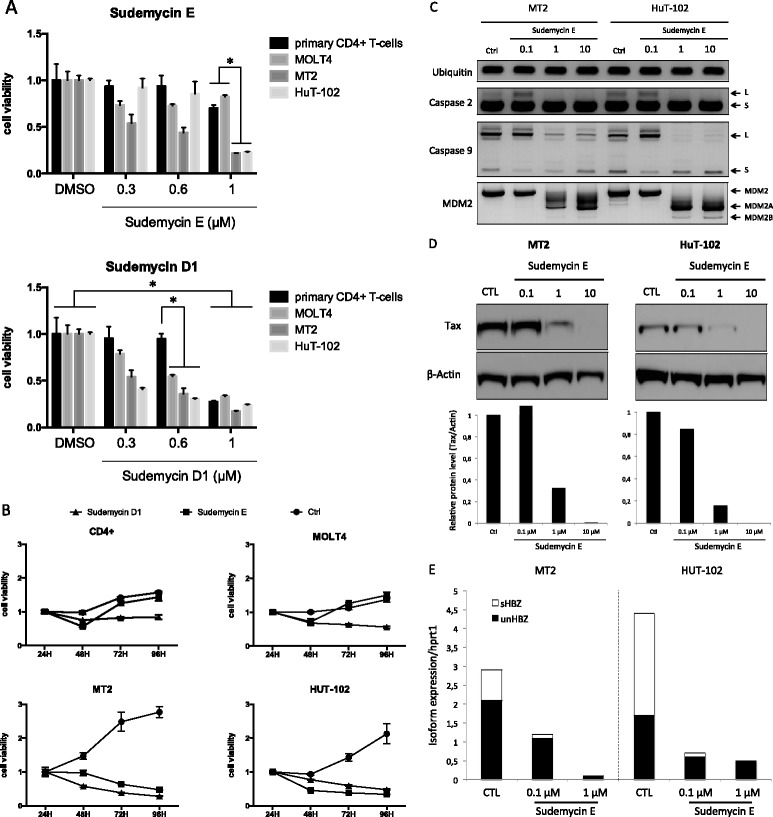
**Anti-proliferative effect of Sudemycin E on HTLV-1-transformed cell lines. (A)** Cell viability was determined by MTT assay (Cell Titer 96® Non-Radioactive Cell Proliferation Assay, Promega) 72 h after Sudemycin (D1 or E) exposure. Data represent normalized mean ± SD; *p < 0.05 was considered statistically significant using a Mann–Whitney U-test. **(B)** *Time-course analysis over 96 h using 1 μM of Sudemycin.* Experiments were performed twice, in triplicate for each cell type, with DMSO used as control. Data represent the normalized mean ± SD. **(C)** *Sudemycin E-induced expression of short spliceoforms encoding caspases 2 and 9 and MDM2.* Exon-specific RT-PCR was carried out 72 h after Sudemycin E exposure. The oligonucleotides used have been previously described [36]. **(D)** *Sudemycin E reduces Tax expression.* Western blot analysis of *Tax* expression was carried out with a mouse monoclonal anti-Tax antibody (clone 474) 72 h after Sudemycin E exposure. Histograms represent the quantification of Tax signals normalized against beta-actin. **(E)** *Sudemycin E decreases gene expression level of HBZ and the relative ratio sHBZ/unHBZ.* Isoform-specific qRT-PCR analysis was carried out after 72 h of Sudemycin exposure. HBZ isoform expressions were normalized to *hprt-1* transcripts used as internal control.

The plethora of splicing events found in transformed and untransformed-infected cells suggests that spliceosome components might constitute new therapeutic targets in the management of HTLV-1 infection. Thus, we examined whether the spliceosome modulators Sudemycin E and D1 [36] exerted anti-proliferative activity against HTLV-1 T-cell lines. To this end, cell viability upon drug exposure was compared between HTLV-1-transformed cell lines (MT2 and HUT-102) and uninfected cells, including primary CD4^+^ T-cells and the HTLV-1-negative acute lymphoblastic leukemia cell line, MOLT4. Figure 3A shows that increasing the concentration of Sudemycin D1 led to a rapid decrease in the viability of human cell lines whether they carried HTLV-1 proviruses or not, while growth of primary CD4^+^ T-cells was inhibited at higher drug concentrations. In contrast, a similar Sudemycin E concentration range was found to significantly decrease the viability of HTLV-1 cell lines, while displaying weak effects on primary CD4^+^ and MOLT4 cells. High concentration of Sudemycin E (>5 μM) affected cell viability of all tested cells, carrying or not HTLV-1 (not shown). These data suggest that, compared to both transformed and untransformed-uninfected cells, HTLV-1 tumoral cells are highly sensitive to drugs that target splicing machinery. Time-course analysis confirmed these results and further revealed that the anti-proliferative effect of Sudemycin E was persistent in MT2 and HUT-102 HTLV-1-positive cells relative to HTLV-1-negative CD4^+^ and MOLT4 cells (Figure 3B).

Further analyses examined the impact of Sudemycin E on the expression of viral and cellular genes. Figure 3C shows that Sudemycin E dose-dependently induced the expression of short spliceoforms of MDM2 and caspases 2 and 9 that have been previously correlated with apoptosis induction [37,38]. In contrast, the expression of ubiquitin, resulting from constitutive splicing of intron 3, remained unaffected. This indicates that Sudemycin E only affected alternative splicing, rather than whole gene transcription, of apoptotic regulatory genes. In parallel, as Tax is synthetized by double-spliced viral transcripts, we examined whether Sudemycin E could affect Tax protein levels. As shown in Figure 3D, western blot analysis of MT2 and HuT102 cell lines revealed that increasing the concentration of Sudemycin E led to reduced Tax expression. Because ATLL cells barely express Tax but regularly show high levels of HBZ mRNA, we next aimed at testing the effects of Sudemycin E on HBZ expression. In contrast to Tax, HBZ expression is not strictly dependent on splicing events. In fact, HTLV-1 minus strand encodes spliced (sHBZ) and unspliced isoforms (unHBZ) of HBZ, yet sHBZ is the most abundant in HTLV-1 transformed cell lines and in fresh ATLL samples [20,39]. In contrast to its unspliced counterpart, sHBZ promotes cell proliferation *in vitro* thereby likely explaining the positive correlation between sHBZ expression and HTLV-1 proviral loads *in vivo* [21,39]. These data prompted us to assess the expression levels of both sHBZ and usHBZ RNA isoforms in Sudemycin E exposed cells using exon-specific quantitative RT-PCR analysis. The results illustrated in the Figure 3E show that both MT2 and HUT102 cells exhibited reduced ratios of sHBZ/unHBZ upon Sudemycin E together with a dramatic decrease in the overall level of unHBZ expression, which is consistent with the transactivating functions of sHBZ on HBZ gene expression [40]. Given the crucial role of both Tax and sHBZ in conferring proliferative and anti-apoptotic properties, these data help explain the heightened sensitivity of HTLV-1-infected cells to the anti-proliferative properties of Sudemycin E compared to their uninfected counterparts. Taken together, our results indicate that by impairing splicing and, in turn, gene expression of HTLV-1 oncogenes, splicing modulators such as Sudemycin E might constitute promising tools for ATLL treatment.

## Conclusions

Our investigation revealed that HTLV-1 significantly qualitatively modifies gene transcripts, as well as unmasks new putative leukemogenic pathways and possible therapeutic targets. Because alternative splicing can result in mRNA isoforms coding for proteins with different biological activity, our results suggest that excessive splicing alterations in HTLV-1-positive cells might underlie their phenotypic plasticity. Future studies are needed to address the molecular mechanisms underlying HTLV-1-induced AEU and their involvement in clonal persistence, immune escape, and/or cellular transformation of infected CD4^+^ cells, as well as their putative role in treatment resistance and relapse of ATLL.

## Methods

### T cell limiting cloning

Peripheral blood mononuclear cells (PBMCs) were obtained by Ficoll separation of whole blood of HTLV-1 infected individuals. For T-cell limiting dilution cloning, PBMCs were seeded at 0.1 cell/well in Terasaki plates after removal of adherent cells. T-lymphocytes were cultured in RPMI 1640 containing penicillin and streptomycin, sodium pyruvate, non-essential amino acids, 2-mercaptoethanol, 10% filtered human AB serum, 100 U/mL recombinant IL-2 (Chiron Corporation), and 75 μM HTLV-1 integrase inhibitor L-731,988 [30]. T-lymphocytes were re-stimulated every 14 days with PHA (1 μg/mL) and fresh feeder cells (5.10^5^ cells/mL) that were composed of lethally irradiated PBMCs from three distinct allogenic HTLV-1-negative donors. Clones were phenotyped by flow cytometry using antibodies against CD4 and CD8 (DakoCytomation) and isotype-matched controls on a FACScan system using CellQuest software (Becton Dickinson). HTLV-1-positive clones were assessed by PCR using long-terminal repeat region–specific primers as previously described [29]. For each clone, RNA extraction was performed before and 24 h after PHA stimulation.

### Microarray analysis

Affymetrix exon array hybridization was completed by labeling 1 μg of TRIzol-purified total RNA with Affymetrix reagents according to the manufacturer’s instructions. Hybridization cocktails containing 5–5.5 μg cDNA were prepared and hybridized to Affymetrix-GeneChip Human Exon 1.0 ST arrays (Affymetrix). Affymetrix Expression Console Software was used for quality assessment, while exon array data were normalized using quintile normalization and analyzed using FasterDB annotation (https://fasterdb.lyon.unicancer.fr/) [41]. Background correction and probe selection were performed as described previously [42]. Statistical analyses were performed using a Student’s *t*-test on the splicing index (SI) that corresponds to comparison of gene-normalized exon intensity values between two experimental conditions [30,42]. According to exon-specific RT-PCR (Figures 1C and D), microarray data were considered statistically significant for p < 0.05 and SI ≥ 1.2.

### Polymerase chain reaction

PCR was performed using the Herculase II Fusion DNA Polymerase (Agilent Technologies) in 30–35 cycles. PCR products were analyzed by agarose gel electrophoresis with ethidium bromide under UV light. Primers were designed to encompass alternative splicing events. Primer sequences are available upon request.

### Quantitative RT-PCR

Expression of *Tax* mRNA was quantified with Rotor Gene (Qiagen) as previously described [29,43]. Expression of sHBZ and unHBZ was quantified using primers previously described by Murata *et al.* [44]. qRT-PCR was performed in triplicate and relative quantitation (RQ) was calculated by the 2ddCT method to normalize gene expression to the endogenous control *U6* (ENSG00000206625) or *hprt1* (ENSG00000165704).

### Drug exposure and cell viability

Cell viability was determined by MTT assay (Cell Titer 96® Non-Radioactive Cell Proliferation Assay, Promega) 72 h after Sudemycin (D1 or E) exposure. The MTT dye was added during the last hour of incubation. After, supernatants were removed and cells were incubated with 100 μL/well Solubilization Solution/Stop Mix for 1 h at room temperature. The OD was measured using an ELISA reader at 570 nm, with 650 nm as a reference. Experiments were completed twice, in triplicate for each cell type, with DMSO used as control.

## Additional files

Additional file 1: Table S1. List of samples. Additional file 2: Figure S1.
*Tax* expression in infected clones and ATLL samples. Tax mRNA was quantified by real-time qRT-PCR as described previously [29,43]. *Statistically significant differences by Mann–Whitney U-test. Additional file 3: Figure S2. qRT-PCR confirmation of overall gene expression. cDNA was amplified by qRT-PCR in 20 μl reactions using a QuantiFast SYBR Green PCR Kit (Qiagen) with 10 μM of each primer. Reactions were run on a Rotor-Gene 3000 (Corbett Life Science, Australia). HRPT1 gene expression was used as an internal control. A melting curve (57–95°C) was generated at the end of each run to verify primer specificity. The Pfaffl method was used for relative quantification. Primer sequences are available upon request. FC, indicates microarray-predicted positive or negative fold-changes in gene expression between infected and uninfected CD4^+^ cells; CD4UI, uninfected CD4^+^ clones; CD4I, infected CD4^+^ clones. Additional file 4: Table S2. Common AEUs between untransformed-infected CD4^+^ clones and ATLL samples (compared with uninfected clones). Additional file 5: Table S3. GO terms enriched by genes with common AEUs in untransformed-infected clones and ATLL samples (compared with uninfected clones). Additional file 6: Table S4. GO terms enriched by genes displaying AEUs that discriminate between uninfected and infected clones and ATLL samples. Additional file 7: Figure S3. GO analysis in ATLL samples and untransformed clones according to whole gene expression and AEU. The Venn-diagrams show the distribution of genes modified at the whole gene expression level (white; FC ≥ 1.2; p < 0.05), AEU level (dark grey; SI ≥ 1.2; p < 0.05), or both (light grey). Each gene set was analyzed using the DAVID browser (http://david.abcc.ncifcrf.gov/; KEGG pathways). Each gene set was limited to 2000 genes that displayed the highest fold-change in expression and splicing index (SI) values. The complete set of genes featured in microarrays was used as reference background. Top pathways are presented.
