# Supplementary figures and images for: HTLV-1-infected CD4+ T-cells display alternative exon usages that culminate in adult T-cell leukemia
Source: Retrovirology. 2014 Dec 18;11:119. doi: 10.1186/s12977-014-0119-3 (PMC4293115; doi:10.1186/s12977-014-0119-3)

## Slide 1
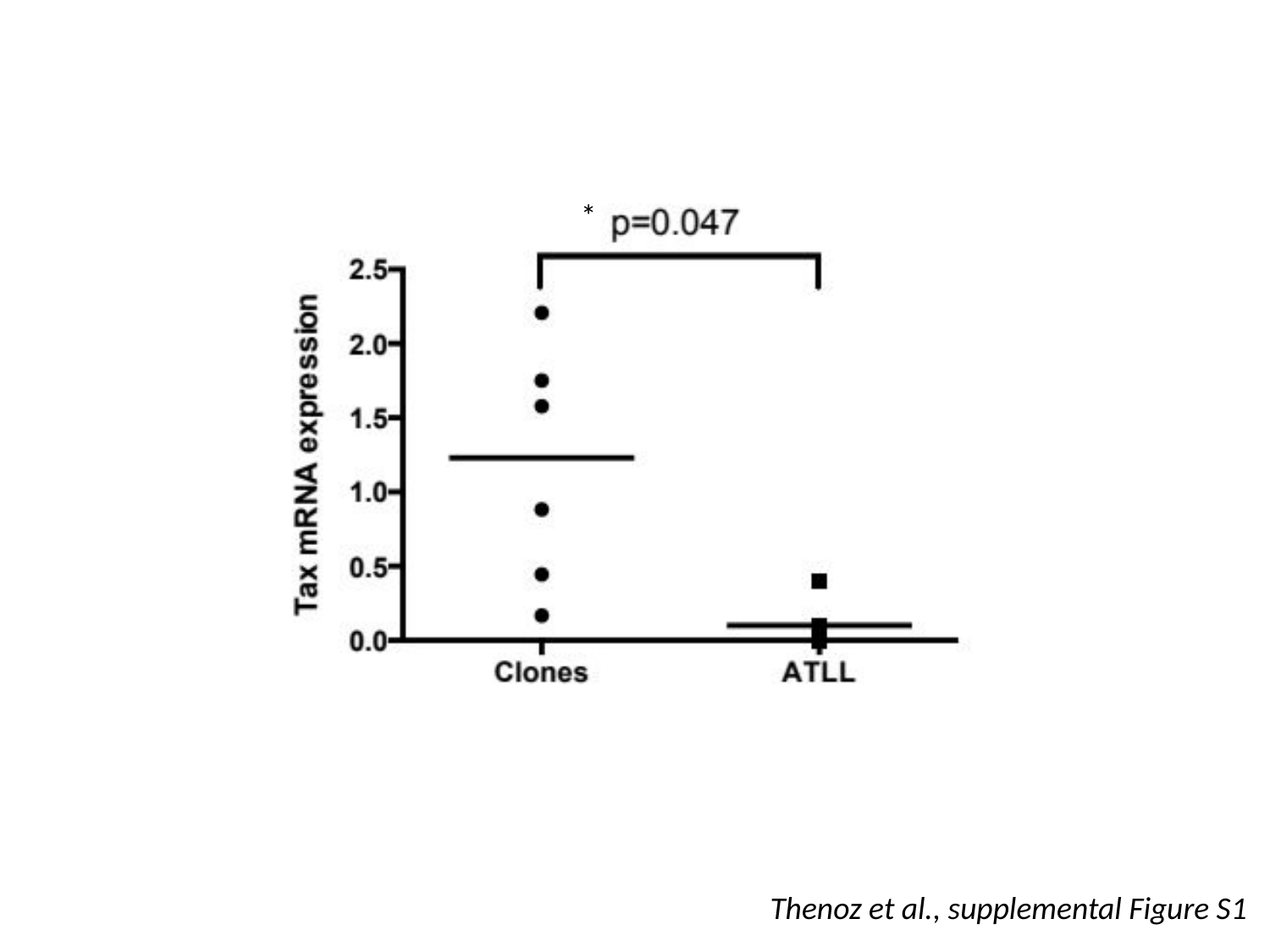

*
Thenoz et al., supplemental Figure S1

Supplement: Additional file 2: Figure S1. — Tax expression in infected clones and ATLL samples. Tax mRNA was quantified by real-time qRT-PCR as described previously [29,43]. *Statistically significant differences by Mann–Whitney U-test. [file 12977_2014_119_MOESM2_ESM.pptx]
