# Supplementary material for: HTLV-1-infected CD4+ T-cells display alternative exon usages that culminate in adult T-cell leukemia
Source: Retrovirology. 2014 Dec 18;11:119. doi: 10.1186/s12977-014-0119-3 (PMC4293115; doi:10.1186/s12977-014-0119-3)
Supplement: Additional file 3: Figure S2. — qRT-PCR confirmation of overall gene expression. cDNA was amplified by qRT-PCR in 20 μl reactions using a QuantiFast SYBR Green PCR Kit (Qiagen) with 10 μM of each primer. Reactions were run on a Rotor-Gene 3000 (Corbett Life Science, Australia). HRPT1 gene expression was used as an internal control. A melting curve (57–95°C) was generated at the end of each run to verify primer specificity. The Pfaffl method was used for relative quantification. Primer sequences are available upon request. FC, indicates microarray-predicted positive or negative fold-changes in gene expression between infected and uninfected CD4+ cells; CD4UI, uninfected CD4+ clones; CD4I, infected CD4+ clones. [file 12977_2014_119_MOESM3_ESM.pptx]

## Slide 1
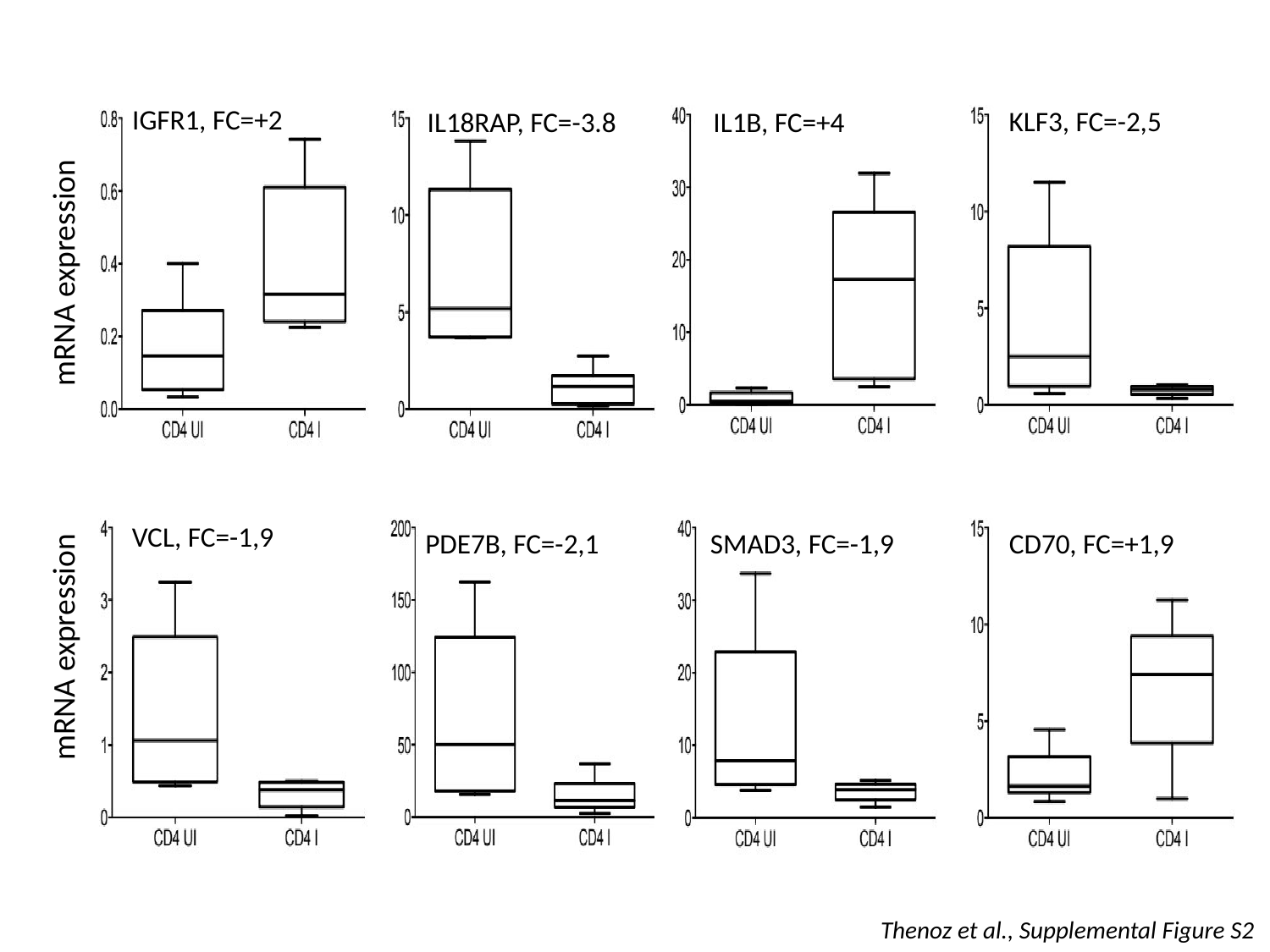

IGFR1, FC=+2
KLF3, FC=-2,5
IL18RAP, FC=-3.8
IL1B, FC=+4
mRNA expression
VCL, FC=-1,9
PDE7B, FC=-2,1
SMAD3, FC=-1,9
CD70, FC=+1,9
mRNA expression
Thenoz et al., Supplemental Figure S2
