# Supplementary material for: HTLV-1-infected CD4+ T-cells display alternative exon usages that culminate in adult T-cell leukemia
Source: Retrovirology. 2014 Dec 18;11:119. doi: 10.1186/s12977-014-0119-3 (PMC4293115; doi:10.1186/s12977-014-0119-3)
Supplement: Additional file 7: Figure S3. — GO analysis in ATLL samples and untransformed clones according to whole gene expression and AEU. The Venn-diagrams show the distribution of genes modified at the whole gene expression level (white; FC ≥ 1.2; p < 0.05), AEU level (dark grey; SI ≥ 1.2; p < 0.05), or both (light grey). Each gene set was analyzed using the DAVID browser (http://david.abcc.ncifcrf.gov/; KEGG pathways). Each gene set was limited to 2000 genes that displayed the highest fold-change in expression and splicing index (SI) values. The complete set of genes featured in microarrays was used as reference background. Top pathways are presented. [file 12977_2014_119_MOESM7_ESM.pptx]

## Slide 1
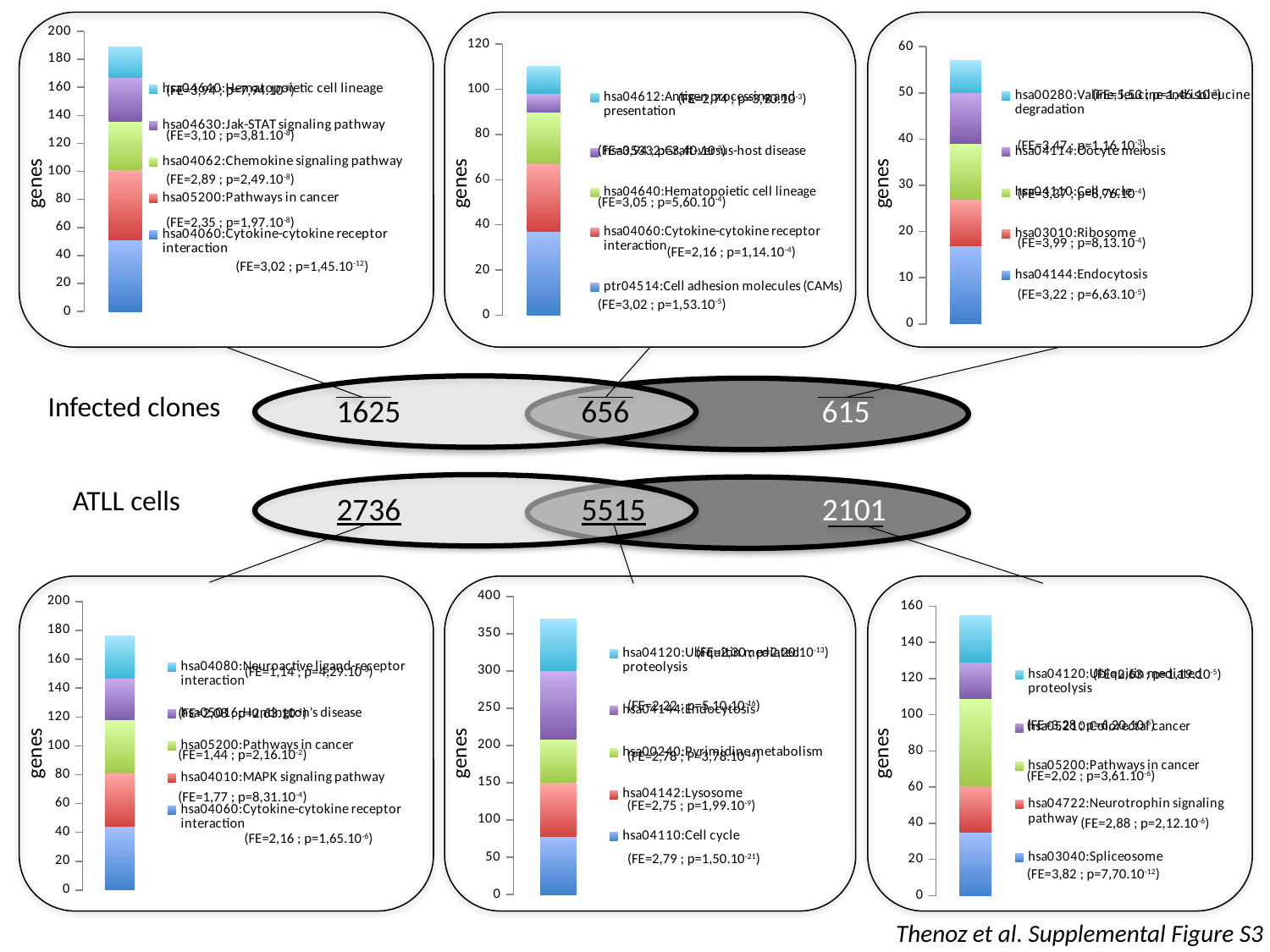

### Chart
| Category | hsa04060:Cytokine-cytokine receptor interaction | hsa05200:Pathways in cancer | hsa04062:Chemokine signaling pathway | hsa04630:Jak-STAT signaling pathway | hsa04640:Hematopoietic cell lineage |
|---|---|---|---|---|---|
### Chart
| Category | ptr04514:Cell adhesion molecules (CAMs) | hsa04060:Cytokine-cytokine receptor interaction | hsa04640:Hematopoietic cell lineage | hsa05332:Graft-versus-host disease | hsa04612:Antigen processing and presentation |
|---|---|---|---|---|---|
### Chart
| Category | hsa04144:Endocytosis | hsa03010:Ribosome | hsa04110:Cell cycle | hsa04114:Oocyte meiosis | hsa00280:Valine, leucine and isoleucine degradation |
|---|---|---|---|---|---|(FE=3,94 ; p=7,94.10-8)
(FE=5,53 ; p=1,46.10-3)
(FE=2,74 ; p=3,90.10-3)
(FE=3,10 ; p=3,81.10-8)
(FE=3,47 ; p=1,16.10-3)
(FE=3,94 ; p=3,40.10-3)
(FE=2,89 ; p=2,49.10-8)
genes
genes
genes
(FE=3,37 ; p=8,76.10-4)
(FE=3,05 ; p=5,60.10-4)
(FE=2,35 ; p=1,97.10-8)
(FE=3,99 ; p=8,13.10-4)
(FE=2,16 ; p=1,14.10-4)
(FE=3,02 ; p=1,45.10-12)
(FE=3,22 ; p=6,63.10-5)
(FE=3,02 ; p=1,53.10-5)
1625
656
615
Infected clones
2736
5515
2101
ATLL cells
### Chart
| Category | hsa04110:Cell cycle | hsa04142:Lysosome | hsa00240:Pyrimidine metabolism | hsa04144:Endocytosis | hsa04120:Ubiquitin mediated proteolysis |
|---|---|---|---|---|---|
### Chart
| Category | hsa04060:Cytokine-cytokine receptor interaction | hsa04010:MAPK signaling pathway | hsa05200:Pathways in cancer | hsa05016:Huntington's disease | hsa04080:Neuroactive ligand-receptor interaction |
|---|---|---|---|---|---|
### Chart
| Category | hsa03040:Spliceosome | hsa04722:Neurotrophin signaling pathway | hsa05200:Pathways in cancer | hsa05210:Colorectal cancer | hsa04120:Ubiquitin mediated proteolysis |
|---|---|---|---|---|---|(FE=2,30 ; p=2,20.10-13)
(FE=1,14 ; p=4,29.10-3)
(FE=2,63 ; p=1,19.10-5)
(FE=2,22 ; p=5.10.10-16)
(FE=2,08 ; p=2,63.10-4)
(FE=3,28 ; p=6,20.10-6)
genes
genes
genes
(FE=1,44 ; p=2,16.10-2)
(FE=2,78 ; P=3,78.10-16)
(FE=2,02 ; p=3,61.10-6)
(FE=1,77 ; p=8,31.10-4)
(FE=2,75 ; p=1,99.10-9)
(FE=2,88 ; p=2,12.10-6)
(FE=2,16 ; p=1,65.10-6)
(FE=2,79 ; p=1,50.10-21)
(FE=3,82 ; p=7,70.10-12)
Thenoz et al. Supplemental Figure S3
